# Leukocyte Ratios Predict Metastasis, Recurrence, and Mortality in Breast Cancer Patients Receiving Cytotoxic Chemotherapy

**DOI:** 10.3390/medsci13040285

**Published:** 2025-11-26

**Authors:** Carolina Coradi, Aedra Carla Bufalo Kawassaki, Ana Paula Vieira, Camila Elizandra Rossi, Caryna Eurich Mazur, Claudiceia Risso Pascotto, Cleide Viviane Buzanello, Dalila Moter Benvegnú, Franciele Aní Caovilla Folador, Geraldo Emílio Vicentini, Gisele Arruda, Guilherme Welter Wendt, Kérley Braga Pereira Bento Casaril, Léia Carolina Lucio, Lirane Elize Defante Ferreto, Mariana Abe Vicente Cavagnari, Tatiane Renata Fagundes, Daniel Rech, Carolina Panis

**Affiliations:** 1 Laboratory of Tumor Biology, State University of West Paraná, UNIOESTE, Francisco Beltrão 85601-970, Parana, Brazil; carolina_coradi@hotmail.com (C.C.); tatiane.fagundes3@hotmail.com (T.R.F.);; 2 Health Sciences Post-Graduation Program, University of West Paraná, UNIOESTE, Francisco Beltrão 85601-970, Parana, Brazil; aedrab@gmail.com (A.C.B.K.); dalila.benvegnu@uffs.edu.br (D.M.B.); franciele.follador@unioeste.br (F.A.C.F.); geraldo.vicentini@unioeste.br (G.E.V.); kerley.casaril@unioeste.br (K.B.P.B.C.);; 3 Experimental Pathology Post-Graduation Program, State University of Londrina, UEL, Londrina 86057-970, Parana, Brazil; 4 Department of Biological Sciences, State University of North Paraná, UENP, Bandeirantes 86360-000, Parana, Brazil

**Keywords:** breast cancer, hematological ratios, biomarkers, prognosis

## Abstract

**Background/Objectives**: Breast cancer is a leading cause of female mortality worldwide. Immune system dynamics, reflected in hematological ratios derived from leukocytes, have been increasingly recognized for their prognostic value in cancer patients. This study aimed to evaluate the clinical significance of leukocyte-based hematological ratios in breast cancer patients undergoing cytotoxic polychemotherapy, focusing on their association with prognosis, chemoresistance, recurrence, metastasis, and mortality. **Methods**: A mixed observational study was conducted with 185 breast cancer patients undergoing AC-T chemotherapy. Hematologic ratios, including neutrophil-to-lymphocyte (NLR), monocyte-to-lymphocyte (MLR), and platelet-to-lymphocyte (PLR), were calculated at multiple treatment points (D0–D168) and correlated with clinical outcomes. Statistical analyses included ANOVA and ROC curve evaluations to determine the prognostic accuracy of these markers. **Results:** Significant alterations in hematological ratios were observed during chemotherapy. An increase in MLR correlated with intermediate risk for death and metastasis, while elevated PLR and platelet-to-neutrophil ratio (PNR) were strongly associated with metastasis, recurrence, and mortality. Decreases in lymphocyte-to-platelet ratio (LPR) were linked to chemoresistance and adverse outcomes. ROC curve analysis identified PLR at D84 (sensitivity: 83.33%) and PNR at D126 (specificity: 87.01%) as robust prognostic markers. **Conclusions**: Leukocyte-based hematological ratios provide valuable insights into immune dynamics and prognosis in breast cancer patients undergoing chemotherapy. Their integration into routine clinical evaluations could enhance risk stratification and personalized treatment approaches.

## 1. Introduction

Breast cancer is the most common malignant neoplasm affecting women worldwide and the leading cause of female mortality. According to the World Health Organization (WHO), the disease caused 670,000 deaths globally in 2022 alone, making it a significant global public health concern [1].

An increasingly recognized factor is the role of the immune system in the progression and prognosis of the disease [2,3]. In this context, immune system cells play a fundamental role in cancer surveillance by recognizing tumor antigens and promoting inflammation—a hallmark of cancer that can either eliminate or facilitate tumor progression. The immune system is characterized by its dynamic structure, capable of traversing all tissues and fluids during leukocyte homing. This mobility is reflected in blood composition and holds significant value in clinical practice for the systemic assessment of patient health [4].

The hematopoietic system is essential for maintaining human health and is responsible for the production of immune cells. Different blood cell lineages play unique roles, such as erythrocytes, which are responsible for oxygen transport; platelets, which prevent bleeding; and leukocytes, which fight infections, maintain the healing and repair process, and perform immunosurveillance against tumors and autoimmunity [5].

The process of renewal of these cells is called hematopoiesis, characterized by the production of new blood cells generated from hematopoietic stem cells. These cells are primarily found in the bone marrow and are characterized by their self-renewal capacity [6,7]. From hematopoietic stem cells, the formation of multipotent progenitors occurs, which differentiate into myeloid and lymphoid progenitors. The myeloid progenitor is responsible for the differentiation of cells such as erythrocytes, platelets, and granulocytes, including neutrophils, monocytes, and eosinophils. The lymphoid progenitor, on the other hand, gives rise to T cells, B cells, and natural killer (NK) cells [5].

In recent years, studies have used this information to predict outcomes in breast cancer based on the counts and proportions of immune cells, calculated from the patients’ hematological data [8]. Among the most commonly evaluated are the neutrophil-to-lymphocyte ratio (NLR) and platelet-to-lymphocyte ratio (PLR), which have been recognized as indicators of systemic inflammation in various cancers [9]. These markers have been associated with disease-free survival (DFS) and overall survival (OS) rates [10,11,12], as well as being suggested as predictors of metastasis [13] for breast cancer patients. Despite their relevance, existing data are based on single-point analyses and do not account for the predictive and prognostic value of leukocyte fluctuations throughout cytotoxic treatment. Considering the potential of these indices as adjuvant markers for monitoring breast cancer treatment, this study evaluated the clinical significance of leukocyte-based hematological ratios in breast cancer patients undergoing polychemotherapy during the first treatment cycle, with a focus on treatment responsiveness, disease progression, and patient survival.

## 2. Materials and Methods

### 2.1. Study Design

**Figure 1:**
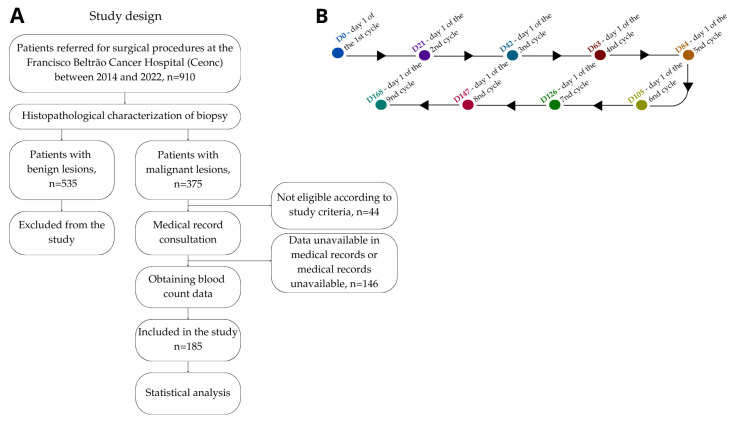
Study design (**A**) and chemotherapy timeline (**B**). Each point on the timeline (marked with “D” followed by a number) represents the start of a new treatment cycle and hemogram data collection. The arrows indicate the progression between the data collection cycles.

This is a mixed observational study, with both qualitative and quantitative approaches, retrospective and prospective, approved by the Institutional Ethics Committee (CAAE number 35524814.4.0000.0107). A total of 910 patients were invited to participate, all referred for surgical procedures at the Cancer Hospital of Francisco Beltrão (Ceonc, Cascavel, Brazil) with lesions suggestive of unilateral invasive ductal carcinoma (IDC, Needham, MA, USA) at any clinical stage. After signing the informed consent form, tissue samples from the biopsies were histopathologically categorized based on the presence of IDC or benign lesions by a pathologist. Exclusion criteria included: patients with benign lesions (*n* = 535); patients with unavailable data in their medical records (*n* = 146); patients who underwent fewer than 4 cycles of chemotherapy (*n* = 44). Inclusion criteria included: patients with malignant breast lesions; patients who underwent more than 4 cycles of chemotherapy; patients with available hemogram data in their medical records. A total of 185 patients were included (Figure 1A).

The treatment regimen used by the evaluated patients was AC-T. This chemotherapy scheme consists of the sequential administration of two drugs (Doxorubicin and Cyclophosphamide) for three weeks, followed by 12 weekly injections of Paclitaxel, with the following doses: Doxorubicin (Adriamycin) 60 mg/m^2^, administered intravenously (IV) every 3 weeks; Cyclophosphamide (Cytoxan) 600 mg/m^2^, IV, every 3 weeks; Paclitaxel (Taxol) 80 mg/m^2^, IV, weekly for 12 weeks. The initial phase with Doxorubicin and Cyclophosphamide lasted 4 cycles (every 3 weeks), followed by the weekly Paclitaxel phase for 12 weeks. In HER2-positive patients, the following scheme was adopted: loading dose (initial) of 8 mg/kg body weight, administered IV over 90 min. Maintenance dose (after the loading dose) of 6 mg/kg body weight, administered IV every 3 weeks. The infusion was administered over 30 min for 12 months. All ER/PR-positive patients also received treatment with tamoxifen or anastrozole. Tamoxifen regimen: 20 mg per day, administered orally for 5 to 10 years; anastrozole regimen: 1 mg per day, administered orally for 5 to 10 years [14].

### 2.2. Hemogram Data Collection

Medical records were reviewed to obtain hemogram data. Peripheral blood samples (5–10 mL) were collected in EDTA tubes and analyzed for complete blood count at a laboratory affiliated with the hospital. Hemogram data were collected on D0, before the start of chemotherapy treatment, and every 21 days (D0, D21, D42, D63, D84, D105, D126, D147, D168) (Figure 1B). Each 21-day period corresponds to one chemotherapy treatment cycle. The following parameters were obtained: differential count of lymphocytes, monocytes, neutrophils, and platelet count. The medical record was consulted to identify the dates of chemotherapy administration at the hospital. Based on these dates, the corresponding hemograms were selected for pairing. Hemograms taken up to seven days before each treatment cycle were considered.

Patients were followed throughout the chemotherapy regimen, with hematological data collected every 21 days, corresponding to each treatment cycle. Not all patients completed all cycles sequentially, as treatment continuation or temporary interruption was determined by the attending oncologist according to individual clinical conditions, toxicity, or therapeutic response. These variations did not represent loss to follow-up but rather medical decisions inherent to personalized chemotherapy protocols. Consequently, hematological ratios were analyzed longitudinally, including all available data points for each patient within the follow-up period.

### 2.3. Collection of Clinical-Pathological Data

Clinical data obtained during the medical consultation and from the medical record were used to gather the following items: age at diagnosis (categorized as below or above 50 years), molecular subtype, and TNM staging. Based on these observations, the treatment response profile was determined (responsive or chemoresistant to the first treatment cycle according to the RECIST 11.0 Guideline [15]), risk stratification for death and recurrence (low, intermediate, and high), and the pattern of disease spread in metastases (absence or presence) [16].

### 2.4. Receiver Operating Characteristic (ROC) Curves

For the construction of the ROC curves, GraphPad Prism 9.0 (GraphPad Software, San Diego, CA, USA) was used. The data were organized into tables with two main columns: the first containing the hematological ratios for the groups without the evaluated outcome, and the second containing the hematological ratios with the positive outcome. Sensitivity was represented on the Y-axis and 1-specificity on the X-axis. The test was performed using the Wilson/Brown method, with a 95% confidence interval, and the results were presented as percentages. Subsequently, the Youden Index (sensitivity + specificity − 1) was calculated in Microsoft Excel (https://microsoft.com/excel, accessed on 10 March 2025) to determine the best cutoff point [17].

### 2.5. Statistical Analysis

The results of the hemograms from 185 patients were collected and entered into Excel software (https://microsoft.com/excel, accessed on 10 March 2025) to create groups and comparison categories based on the clinical-pathological profile of the patients included in this study. Hematological ratios of the obtained parameters were then calculated in Excel software. For data analysis, multiple comparisons from D0 to D168 were performed, comparing all hemogram parameters obtained each month, using paired one-way ANOVA for repeated measures, followed by Tukey’s post hoc test. A significance level of *p* < 0.05 was considered. All statistical analyses were conducted in GraphPad Prism version 9.0 software. The following criteria were adopted for inclusion in the final version of this study: a 50% variation between the reference point (D0) and other treatment days; comparisons between D0 and other treatment days (D0 vs. D21, D42, D63, D84, D105, D126, D147, D168); data related to disease prognosis groups such as risk stratification for death and recurrence, chemoresistance, recurrence, metastasis, and death.

## 3. Results

The clinicopathological characteristics of the study population are shown in Table 1. Approximately 39.46% were under 50 years of age, while 59.46% were 50 years or older. Regarding molecular subtype, 28.65% were classified as luminal A, 37.30% as luminal B, 11.89% as HER2-amplified, and 20.00% as triple-negative. In the risk stratification for death and recurrence, 4.86% of the patients were considered low risk, 52.97% intermediate risk, and 38.92% high risk. Regarding chemoresistance, 60.00% of the patients did not exhibit resistance to chemotherapy, while 23.78% demonstrated resistance.

Concerning disease recurrence, 78.92% of the patients did not experience recurrence, 9.19% had recurrence, and 11.89% did not have this information available. About metastasis, 43.24% of the patients did not have metastasis, 42.16% were positive for metastasis, and 14.59% did not have this information reported. Finally, 92.97% of the patients were alive at the time of the evaluation, 5.95% had died previously, and 1.08% did not have this information recorded.

Regarding the evaluation of cell ratios, most of the variations correspond to substantial increases in cell proportions. These variations may indicate changes in the immune profile of patients throughout the treatment, suggesting possible correlations between these alterations and clinical outcomes. The following tables present only the data with statistical significance among the ratios and clinical-pathological parameters analyzed (*p* < 0.05).

In the group of patients with intermediate risk for death and recurrence (Table 2), a significant increase was observed in the MON/LYM ratio, which showed a 59% rise at 63 days of treatment (95% CI: −0.1756 to −0.005648, *p* = 0.0211). The MON/NEU ratio also increased by 101% at 63 days (95% CI: −0.1453 to −0.03128, *p* < 0.0001) and by 69% at 84 days (95% CI: −0.1142 to −0.007543, *p* = 0.0072). In contrast, the MON/PLT ratio significantly decreased, with a 51% reduction at 126 days (95% CI: 4.656 × 10^−5^ to 0.001317, *p* = 0.0192), while the NEU/PLT ratio decreased by 55% during the same period (95% CI: 0.004264 to 0.01344, *p* < 0.0001). On the other hand, the PLT/NEU ratio increased substantially, with a 102% rise at 105 days (95% CI: −132.3 to −9.958, *p* = 0.0052).

Among the patients with chemoresistance (Table 3), a significant reduction was identified in the LYM/PLT ratio, which decreased by 52% at 105 days (95% CI: 0.002784 to 0.007354, *p* < 0.0001). Similarly, the MON/PLT ratio showed a 55% reduction during the same period (95% CI: 0.0001606 to 0.001579, *p* = 0.0026).

In the group of patients with distant metastases (Table 4), significant increases were observed in several hematological ratios. The MON/LYM ratio increased by 59% at 63 days (95% CI: −0.1819 to −0.006787, *p* = 0.02). The PLT/LYM ratio showed a 65% rise at 42 days (95% CI: −141.2 to −23.20, *p* = 0.0002), while the PLT/NEU ratio registered an increase of 111% at 21 days (95% CI: −138.3 to −14.27, *p* = 0.0025) and 107% at 126 days (95% CI: −138.8 to −8.449, *p* = 0.0102).

In the subgroup of patients with a positive outcome for death (Table 5), the main change observed was a significant reduction in the LYM/PLT ratio, which decreased by 53% at 105 days (95% CI: 0.0003443 to 0.009629, *p* = 0.0211). Additional information can be found in the Supplementary Tables (Tables S1–S4).

**Figure 2:**
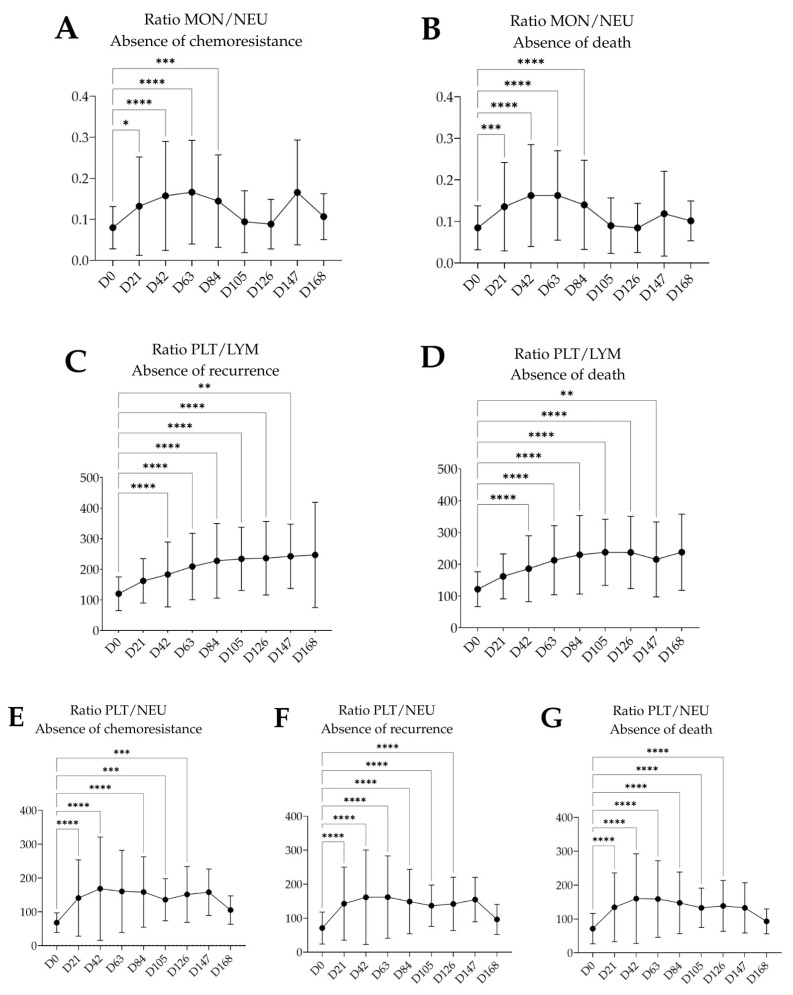
Dynamics of the hematological ratios MON/NEU (**A**,**B**), PLT/LYM (**C**,**D**), and PLT/NEU (**E**–**G**) in association with the absence of chemoresistance, recurrence, and death, respectively. Statistical comparisons were performed throughout the polychemotherapy treatment period. D0 to D168: follow-up days. (* *p* < 0.05; ** *p* < 0.01; *** *p* < 0.001; **** *p* < 0.0001). Abbreviations: MON: monocytes; NEU: neutrophils; PLT: platelets; LYM: lymphocytes.

Figure 2 illustrates the evolution of different hematological parameters throughout the treatment period, as well as comparisons between the different groups. The hematological ratio between monocytes and neutrophils showed a peak increase of 108.5% at D63, followed by a decrease of 13.06% at D84 in the group negative for chemoresistance (Figure 2A). In the group without death, this ratio increased by 91.72% at D63, followed by a 13.84% decrease at D84 (Figure 2B).

The ratio between platelets and lymphocytes showed an increase of 18.61% from D0 to D147 in the group without recurrence (Figure 2C). In the group without death, the same ratio increased by 95.18% from D0 to D126, followed by a slight decrease of 9.24% at D147 (Figure 2D).

Regarding the ratio between platelets and neutrophils, in the group negative for chemoresistance (Figure 2E), there was an increase of 136.19% at D42, followed by a progressive decrease until D105, with a drop of 15.36%. Subsequently, an increase of 11.60% was observed until D126.

In the group without recurrence (Figure 2F), the ratio between platelets and neutrophils increased by 128.18% until D63, followed by a decrease of 8.14% at D84. In the group without death, the same ratio increased by 122.87% until D63, with a reduction of 7.27% at D147 (Figure 2G).

**Figure 3:**
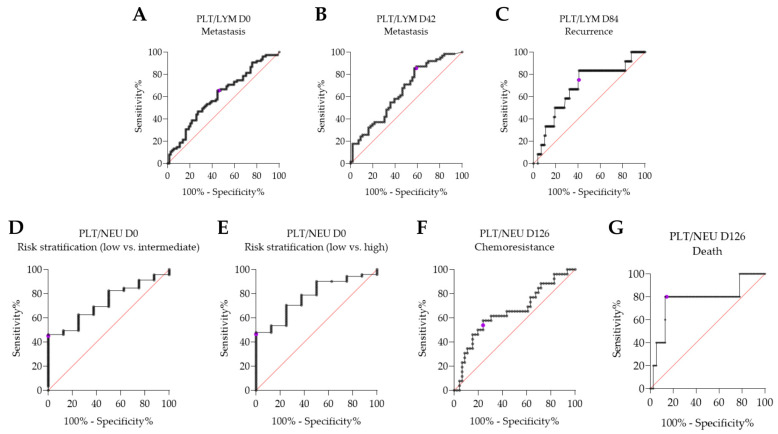
ROC curves illustrating the predictive capacity of the hematological ratios for various outcomes and on different treatment days. (**A**) PLT/LYM for metastasis on day 0 of treatment; (**B**) PLT/LYM for metastasis on day 42; (**C**) PLT/LYM for recurrence on day 84; (**D**) PLT/NEU for risk stratification on day 0; (**E**) PLT/NEU for risk stratification on day 0; (**F**) PLT/NEU for chemoresistance on day 126; (**G**) PLT/NEU for death on day 126. The purple point represents the best cutoff point, calculated using the Youden Index. Abbreviations: “D”: days of follow-up, NEU: neutrophils, PLT: platelets, LYM: lymphocytes.

The results of the ROC (Figure 3 and Table 6) curves showed that in the metastasis group, two hematologic ratios performed well as predictors of outcomes in the ROC curve, with PLT/LYM at D0 showing a cutoff value of 107.7 (sensitivity 65.33, 95% CI 54.05 to 75.12, specificity 55.41, 95% CI 44.09 to 66.18) and PLT/LYM at D42 showing a cutoff value of 130.4 (sensitivity 85.48, 95% CI 74.66 to 92.17, specificity 42.86, 95% CI 30.77 to 55.86). In the recurrence outcome group, the cutoff value of the PLT/LYM ratio at D84 was 181.7, with a sensitivity of 83.33 (95% CI 55.20 to 97.04) and specificity of 59.23 (95% CI 50.64 to 67.30).

In the risk stratification group for death and recurrence, when comparing the low-risk group with the intermediate-risk group, the PLT/NEU ratio at D0 had a cutoff value of 62.08, with sensitivity of 46.15 (95% CI 36.28 to 56.34) and specificity of 100.0 (95% CI 67.56 to 100.0). In the comparison between the low-risk group and the high-risk group, the PLT/NEU ratio at D0 also showed significance, with a cutoff value of 61.68, sensitivity of 47.89 (95% CI 36.68 to 59.31) and specificity of 100.0 (95% CI 67.56 to 100.0).

In chemotherapy resistance group, the hematological ratio PLT/NEU at D126 had a cutoff value of 111.5, with a sensitivity of 57.69 (95% CI 38.95 to 74.46) and specificity of 76.09 (95% CI 62.06 to 86.09). In the group with death, the PLT/NEU ratio at D126 had a cutoff value of 202.1, with sensitivity of 80.00 (95% CI 37.55 to 98.97) and specificity of 87.01 (95% CI 77.72 to 92.79).

## 4. Discussion

Leukocyte-based hematological ratios have been identified as significant markers in the evaluation of cancer patients. In this study, we assessed the utility of these ratios as prognostic biomarkers in breast cancer patients undergoing polychemotherapy during the first treatment cycle. The primary endpoints examined included treatment responsiveness, disease progression, and patient survival. Our results revealed significant changes in cellular proportions throughout treatment, with notable correlations with clinical outcomes. Key changes were observed in the ratios of lymphocytes, neutrophils, monocytes, and platelets, which reflected alterations in the immune profile of patients during treatment.

The clinical response to chemotherapy is highly variable and depends on a complex network of factors involving the treatment, the tumor, and patient characteristics, as well as their multiple interactions. Maintaining the dose intensity over time is essential for therapeutic efficacy; dose reductions or cycle delays, often imposed by myelosuppression, may compromise disease control and patient survival [18]. However, even when the regimen is maintained, tumor resistance—whether intrinsic or acquired—remains a major obstacle. This resistance can manifest through drug efflux mechanisms mediated by transporters and by apoptosis evasion, resulting in a drastic reduction in intracellular concentration and cytotoxic agent efficacy [19].

The tumor microenvironment (TME) plays a central role in this process. Hypoxic conditions and the presence of stromal cells, such as fibroblasts and macrophages, promote the secretion of soluble factors like TGF-β, which support cancer cell survival and resistance. Furthermore, the patient’s clinical and immunological status directly influences treatment tolerance [20,21].

In this context, the immune system can act as both an ally and a saboteur of the treatment. The antitumor response depends on the integrity of effector immunity, but tumor-modulated immune cells often contribute to negative outcomes. Thus, although chemotherapy is designed to directly destroy tumor cells, its ultimate success depends on how it interacts with the host’s biology and the TME [22].

The analysis of hematological ratios, specifically between monocytes and lymphocytes, throughout treatment follow-up was associated with an intermediate risk of death, recurrence, and the development of distant metastases. Furthermore, an increased monocyte-to-neutrophil ratio has been correlated with an intermediate risk of both death and recurrence. This hematological imbalance involving monocytes may reflect the expansion of monocytic myeloid-derived suppressor cells (M-MDSCs), whose proliferation is stimulated during tumorigenesis by the release of factors such as GM-CSF and M-CSF. In peripheral lymphoid tissues, M-MDSCs retain the ability to differentiate into functional macrophages and dendritic cells. However, within the tumor microenvironment, this process is redirected, leading these cells to predominantly convert into tumor-associated macrophages (TAMs). Both TAMs and M-MDSCs secrete cytokines such as IL-6, IL-10, TGF-β, and vascular endothelial growth factor (VEGF), which promote angiogenesis and immune suppression, thereby contributing to the establishment of a pro-inflammatory tumor microenvironment [23,24,25]. On the other hand, the reduction in lymphocyte count in peripheral blood reflects a compromise in adaptive immunity. T lymphocytes, in particular, are crucial in the destruction of tumor cells, and their reduction may be caused by exhaustion of these cells, which lose the ability to attack the tumor, or by suppression mediated by MDSCs [26].

We observed an elevation in the platelet-to-neutrophil ratio, which was associated with the development of distant metastases, disease recurrence, and an intermediate risk of death. Additionally, an increase in the platelet-to-lymphocyte ratio was significantly linked to the onset of distant metastases. Chemotherapy can cause thrombocytopenia, characterized by a decreased platelet count. Recovery of platelets after treatment typically occurs within 2 to 3 weeks, but it may extend over several months. The main risk associated with thrombocytopenia includes an increased chance of bleeding, which can be fatal [27,28]. Platelets play essential roles beyond coagulation. They interact with tumor cells, facilitating the invasion and metastasis processes, as well as releasing pro-tumoral factors like TGF-β and VEGF, which stimulate cancer progression. This may explain the increase in hematological ratios involving platelets [29].

Neutrophils, in turn, promote chronic inflammation and play a key role in tumor progression and metastasis by secreting IL-10, which suppresses the function of cytotoxic T cells and inhibits NK cells, leading to resistance [30]. The platelet-to-lymphocyte ratio was also elevated in patients with metastasis, which can be explained by the combined action of platelets in protecting tumor cells and the inability of lymphocytes to mount an effective immune response [26,29].

The reduction in the lymphocyte-to-platelet ratio over time was associated with chemotherapy resistance and higher mortality among the evaluated patients. Similarly, a decrease in the monocyte-to-platelet ratio was observed in the intermediate-risk groups for death and recurrence and was also linked to chemotherapy resistance. Chemotherapy success depends on adaptive immunity to eliminate tumor cells that evade the drug’s effects. The low count of lymphocytes and monocytes reflects immune exhaustion and a weakened immune response against the tumor, allowing the proliferation of tumor cells that are resistant to treatment [31]. These findings corroborate previous studies suggesting that hematological ratios are predictors of survival in cancer patients [8,32,33]. However, unlike previous studies, this work offers a longitudinal analysis throughout the treatment, emphasizing the dynamics of such specific hematological ratios.

The initial improvement in the immune system, reflected by the increase in hematological ratios during the early treatment cycles (Figure 2), may be linked to immune activation triggered by chemotherapy-induced cytotoxicity on breast tumor cells, followed by the mobilization of immune cells to the tumor site through antigen recognition. During this immune activation period, the release of pro-inflammatory factors such as IL-1β and TNF-α can promote the migration of monocytes, neutrophils, and platelets, enhancing the action of immune cells against the tumor [34,35]. However, as chemotherapy cycles progress, the immune response tends to decline, which can be explained by the impact of polychemotherapy on hematologic tissue, as well as by the state of T cell exhaustion—characterized by the expression of immune checkpoint molecules such as CTLA-4 and PD-1/PD-L1—that reduce their functional capacity [36,37].

Platelet counts typically follow the trends observed in neutrophil levels, and the recovery of platelet counts is considered a sign of good prognosis. Therefore, platelet- and neutrophil-based ratios may serve as valuable markers for evaluating chemotherapy effectiveness throughout treatment cycles. As shown in our ROC curves, the PLT/LYM D84 ratio demonstrated a sensitivity of 83.33% in predicting recurrence, highlighting its effectiveness in identifying patients at higher risk. Similarly, the PLT/LYM D42 ratio exhibited a sensitivity of 85.48% in predicting metastasis, further emphasizing its value in tumor progression outcomes. Regarding death, the PLT/NEU D126 ratio showed a specificity of 87.01%, suggesting its reliability in excluding lower-risk cases.

Additionally, the PLT/NEU D126 ratio for chemoresistance demonstrated a specificity of 76.09%. The PLT/NEU D0 ratio for risk stratification also stood out, with specificity approaching 100%. These findings confirm the relevance of these hematological biomarkers as valuable tools in clinical management, enabling a more targeted approach based on objective data. Assessing parameters commonly used in routine clinical and laboratory settings in breast cancer patients, when correlated with the clinical presentation of the disease, can help detect early therapeutic response failure and, as a result, prevent disease progression [15,38].

Cytotoxic chemotherapy is fundamental in the treatment of various malignancies and, in some cases, it is the only available option. Patients undergoing chemotherapy often exhibit alterations not only in leukocyte counts but also in other rapidly dividing tissues, such as mucosa, intestinal epithelium, and hair follicles [39,40]. By targeting hematopoietic tissue, it causes myelosuppression through the impairment of hematopoietic stem and progenitor cells (HSPCs), resulting in a reduction in erythroid, myeloid, and megakaryocytic lineages [40]. Clinically, this manifests as an increased risk of severe infections, fatigue, and bleeding.

Most chemotherapeutic agents are composed of lipophilic substances that are absorbed by the liver but not easily excreted. As a result, it is common for patients to develop hepatic steatosis, cholestasis, and veno-occlusive disease due to the biotransformation of chemotherapeutic drugs into toxic metabolites [41].

Some agents, such as anthracyclines (doxorubicin) and taxanes (paclitaxel and docetaxel), are commonly used in the treatment of early-stage breast cancer because they reduce the risk of recurrence. However, these drugs can induce both short- and long-term cytotoxicity [42]. The generation of reactive oxygen species (ROS) is common, leading to mitochondrial damage, endoplasmic reticulum stress, and the death of various cell types. This process can induce acute cardiotoxicity, potentially resulting in systolic dysfunction and heart failure [43], reduced renal function, electrolyte loss, and, in some cases, progression to chronic kidney disease [44]. In addition, these agents may cause peripheral neuropathy, clinically manifested as pain, motor dysfunction, and tingling sensations [45]. These effects not only impact the patient’s quality of life but also complicate chemotherapy regimens.

Contemporary oncologic treatment, therefore, aims not only to eliminate the tumor but also to reprogram the microenvironment to restore the patient’s immunological competence. Modern strategies include the repolarization of myeloid cells, such as tumor-associated macrophages (TAMs) and tumor-associated neutrophils (TANs), from pro-tumoral to anti-tumoral phenotypes, as well as the deactivation of immune brakes that prevent effective T-cell activity [46]. Immune checkpoints such as PD-1, PD-L1, and CTLA-4 are physiological mechanisms that prevent autoimmunity but are often exploited by tumors to induce T-cell exhaustion [47,48]. Blocking these checkpoints with monoclonal antibodies—such as ipilimumab (anti-CTLA-4) and nivolumab or atezolizumab (anti-PD-1/PD-L1)—has proven effective in restoring cytotoxic responses and promoting tumor cell death [49]. Strategies involving anti-inflammatory drugs and inhibitors of pro-inflammatory cytokines, such as IL-6, TNF-α, and IL-1β, have also shown promise in remodeling the tumor microenvironment and alleviating immune exhaustion by reducing the chronic inflammation that sustains immunosuppression and tumor growth [50,51].

Chemotherapy has traditionally been viewed as immunosuppressive; however, evidence shows that many chemotherapeutic agents act as positive immunomodulators by promoting the induction of immunogenic cell death (ICD)—a type of cell death characterized by the exposure of calreticulin (CRT) on the cell surface, the extracellular release of adenosine triphosphate (ATP) and the nuclear protein high mobility group box 1 (HMGB1), as well as the subsequent activation of the type I interferon (IFN-I) response. In the same environment, necrosis also occurs, usually uncontrolled and inflammatory. However, regulated forms of necrosis, such as necroptosis and ferroptosis, are recognized for being highly immunogenic. As a result, this process leads to a decrease in FOXP3+ regulatory T cells (Tregs) and an increase in CD8+ T lymphocytes, thereby strengthening the antitumor response [52,53].

Several drugs used in the treatment of breast cancer exhibit positive immunomodulatory potential. Cyclophosphamide and paclitaxel act by reducing Treg cells and promoting macrophage polarization toward the M1 phenotype. Moreover, paclitaxel activates antigen-presenting cells (APCs) and stimulates CD8+ T cells, contributing to the reactivation of antitumor immunity [54].

This interaction between chemotherapy and antitumor immunity helps contextualize the hematological results observed in this study. Leukocyte proportions reflect not only the hematologic toxicity of treatment but also the dynamics of the systemic immune response induced by chemotherapy. Thus, variations in hematologic indices can be interpreted as indirect indicators of immunological activation or suppression throughout the course of treatment.

However, the effectiveness of these therapies directly depends on the patient’s hematologic and immunologic integrity. Chemotherapy-induced anemia (CIA) is one of the most common and clinically relevant complications, affecting quality of life, treatment tolerance, and potentially oncologic prognosis [55]. Its etiology is multifactorial, involving direct myelosuppressive effects on hematopoietic progenitor cells in the bone marrow, as well as mechanisms related to cancer-associated chronic inflammation. Increased hepcidin—induced by pro-inflammatory cytokines such as IL-6—leads to iron sequestration in macrophages and the liver, resulting in functional iron deficiency even in the presence of adequate iron stores [56,57].

This deficiency, in turn, impairs erythropoiesis and weakens the immune response, as iron is essential for the proliferation and effector function of T lymphocytes, macrophages, and NK cells. The hypoxia resulting from anemia further worsens the situation by altering immune cell metabolism and promoting a more aggressive and therapy-resistant tumor microenvironment [58]. Moreover, CIA frequently coexists with neutropenia, increasing susceptibility to infections and often necessitating dose reductions or delays in chemotherapy, thereby decreasing treatment intensity and compromising response rates [57].

In addition to hematologic alterations resulting from chemotherapy, hematologic paraneoplastic syndromes also play a significant role in the dysregulation of hematopoiesis and the modification of hematologic indices. Among them are anemia of chronic disease, pure red cell aplasia, reactive leukocytosis (leukemoid reaction), thrombocytosis, eosinophilia, and basophilia, as well as disseminated intravascular coagulation (DIC), which is often associated with the tumor’s release of procoagulant factors. These alterations directly influence hematologic ratios, as they disrupt hematologic homeostasis [59].

Thus, the preservation of immunologic and hematologic function emerges as a critical component for the success of modern antitumor therapies. Anemia and iron deficiency, by weakening antitumor immunity, may reduce the effectiveness not only of chemotherapy but also of immunotherapies, including checkpoint inhibitors and cell-based therapies, directly impacting survival and disease progression [58].

The estimated recovery time of blood cells depends on several factors, including the patient’s age, the type of chemotherapy used, and the number of cycles administered. For this reason, complete blood count (CBC) monitoring is commonly used to assess patients’ health status, since chemotherapy affects lymphoid and myeloid lineages in distinct ways, significantly impacting the immune system. Consequently, different immune cell populations exhibit varying recovery rates after treatment. In general, innate immune cells tend to regenerate more rapidly than adaptive immune cells [60]. Within the myeloid lineage, the main cells affected are granulocytes—particularly neutrophils—which may take from days to months to recover after chemotherapy administration. This condition often leads to neutropenia, the most severe hematologic toxicity observed in cancer patients, increasing the risk of infections and potential treatment interruption [61].

This study has some limitations, including a modest sample size, limited follow-up time, and the absence of assessment of other markers, such as biochemical evaluation of renal and hepatic function, nutritional status, and anemia, which may influence hematopoiesis and immune responses. Because the duration of chemotherapy and the continuity of cycles were individualized according to clinical judgment, some patients did not complete all treatment cycles consecutively. This variability reflects medical decision-making in clinical practice and should not be interpreted as loss to follow-up. To address this issue, all available hematological data for each patient were included in the longitudinal analysis, regardless of cycle interruptions. Additionally, it was not possible to perform regression or multivariate logistic regression analyses, since the relatively small sample size and the presence of missing or unbalanced data across key prognostic variables reduced the statistical power and model stability. As a result, the current findings are based primarily on univariate analyses, which do not account for potential confounding effects of variables such as age, molecular subtype, and tumor stage. Therefore, these results should be interpreted with caution, and future studies with larger, prospectively collected datasets are needed to confirm whether hematologic ratios such as PLR, PNR, and LNR act as independent prognostic indicators in breast cancer. Despite this, our findings demonstrate, for the first time, the clinical significance of leukocyte-based hematological ratios in breast cancer patients undergoing polychemotherapy.

## 5. Conclusions

In conclusion, this study demonstrates that leukocyte and platelet-based hematological ratios are promising tools for risk stratification and prognostic monitoring in breast cancer patients undergoing polychemotherapy. Significant changes in the analyzed ratios, such as the increase in proportions involving monocytes and platelets and the reduction in those related to lymphocytes, are associated with adverse clinical outcomes such as chemoresistance, metastasis, and death. These findings emphasize the importance of dynamic monitoring of these biomarkers during treatment, contributing to more precise and personalized clinical interventions.

## Figures and Tables

**Table 1:** Clinical-pathological data of the study population.

Clinical-Pathological Data	Total (n)	Total (%)
Age		
Under 50 years old	73	39.46%
Over 50 years old	110	59.46%
Unknown	0	0%
Molecular subtype		
Luminal A	53	28.65%
Luminal B	69	37.30%
HER2 amplified	22	11.89%
Triple Negative	37	20.00%
Unknown	04	2.16%
Risk stratification for death and recurrence		
Low risk	09	4.86%
Intermediate risk	98	52.97%
High risk	72	38.92%
Unknown	06	3.24%
Chemoresistance		
Absence	111	60.00%
Presence	44	23.78%
Unknown	30	16.22%
Recurrence		
Absence	146	78.92%
Presence	17	9.19%
Unknown	22	11.89%
Metastasis		
Absence	80	43.24%
Presence	78	42.16%
Unknown	27	14.59%
Death		
Absence	172	92.97%
Presence	11	5.95%
Unknown	02	1.08%

**Table 2:** Significant hematological ratios according to risk stratification for death and recurrence for the group of breast cancer patients with intermediate risk.

				Tukey’s Test	Bonferroni’s Test
Hematological Ratios	Mean 1	Mean 2	Variation	95% Confidence Interval; *p*-Value	95% Confidence Interval; *p*-Value
MON/LYM D0 vs. MON/LYM D63	0.1528	0.2434	59%	−0.1756 to −0.005648 *p* = 0.0211	−0.1781 to −0.003084 *p* = 0.029
MON/NEU D0 vs. MON/NEU D63	0.08766	0.1759	101%	−0.1453 to −0.03128 *p* < 0.0001	−0.1470 to −0.02956 *p* < 0.0001
MON/NEU D0 vs. MON/NEU D84	0.08766	0.1485	69%	−0.1142 to −0.007543 *p* = 0.0072	−0.1158 to −0.005933 *p* = 0.0089
MON/PLT D0 vs. MON/PLT D126	0.001329	0.000647	−51%	4.656 × 10^−5^ to 0.001317 *p* = 0.0192	2.737× 10^−5^ to 0.001337 *p* = 0.0261
NEU/PLT D0 vs. NEU/PLT D126	0.01616	0.00731	−55%	0.004264 to 0.01344 *p* < 0.0001	0.004125 to 0.01358 *p* < 0.0001
PLT/NEU D0 vs. PLT/NEU D105	69.88	141	102%	−132.3 to −9.958 *p* = 0.0052	−134.1 to −8.113 *p* = 0.0063

Abbreviations: MON: monocytes; LYM: lymphocytes; NEU: neutrophils; PLT: platelets; “D”: day of treatment.

**Table 3:** Significant hematological ratios according to the group of breast cancer patients with chemoresistance.

				Tukey’s Test	Bonferroni’s Test
Hematological Ratios	Mean 1	Mean 2	Variation	95% Confidence Interval; *p*-Value	95% Confidence Interval; *p*-Value
LYM/PLT D0 vs. LYM/PLT D105	0.009684	0.004615	−52%	0.002784 to 0.007354 *p* < 0.0001	0.002713 to 0.007425 *p* < 0.0001
MON/PLT D0 vs. MON/PLT D105	0.001573	0.0007034	−55%	0.0001606 to 0.001579 *p* = 0.0026	0.0001386 to 0.001601 *p* = 0.003

Abbreviations: MON: monocytes; LYM: lymphocytes; PLT: platelets; “D”: day of treatment.

**Table 4:** Significant hematological ratios according to the group of breast cancer patients with distant metastases.

				Tukey’s Test	Bonferroni’s Test
Hematological Ratios	Mean 1	Mean 2	Variation	95% Confidence Interval; *p*-Value	95% Confidence Interval; *p*-Value
MON/LYM D0 vs. MON/LYM D63	0.1588	0.2532	59%	−0.1819 to −0.006787 *p* = 0.02	−0.1846 to −0.004074 *p* = 0.0266
PLT/LYM D0 vs. PLT/LYM D42	125.6	207.8	65%	−141.2 to −23.20 *p* = 0.0002	−143.0 to −21.37 *p* = 0.0002
PLT/NEU D0 vs. PLT/NEU D21	68.93	145.2	111%	−138.3 to −14.27 *p* = 0.0025	−140.3 to −12.34 *p* = 0.0029
PLT/NEU D0 vs. PLT/NEU D126	68.93	142.6	107%	−138.8 to −8.449 *p* = 0.0102	−140.8 to −6.429 *p* = 0.0128

Abbreviations: MON: monocytes; LYM: lymphocytes; NEU: neutrophils; PLT: platelets; “D”: day of treatment.

**Table 5:** Significant hematological ratios for the group of breast cancer patients with a positive outcome for death.

				Tukey’s Test	Bonferroni’s Test
Hematological Ratios	Mean 1	Mean 2	Variation	95% Confidence Interval; *p*-Value	95% Confidence Interval; *p*-Value
LYM/PLT D0 vs. LYM/PLT D105	0.00941	0.004423	−53%	0.0003443 to 0.009629 *p* = 0.0211	0.0002005 to 0.009772 *p* = 0.0281

Abbreviations: LYM: lymphocytes; PLT: platelets; “D”: day of treatment.

**Table 6:** Area under the curve (AUC) with 95% confidence interval (CI) and *p*-value, cut-off value, sensitivity, specificity, and likelihood ratio for significant ROC curves.

Ratio and Outcome	AUC (95% CI; *p*-Value)	Cut-Off Value	Sensitivity% (95% CI)	Specificity% (95% CI)	Likelihood Ratio
PLT/LYM D0 Metastasis	0.6121 (0.5219 to 0.7023; *p* = 0.0182)	>107.7	65.33 (54.05% to 75.12%)	55.41 (44.09% to 66.18%)	1.465
PLT/LYM D42 Metastasis	0.6483 (0.5491 to 0.7475; *p* = 0.0055)	>130.4	85.48 (74.66% to 92.17%)	42.86 (30.77% to 55.86%)	1.496
PLT/LYM D84 Recurrence	0.6830 (0.5246 to 0.8414; *p* = 0.0363)	<181.7	83.33 (55.20% to 97.04%)	59.23 (50.64% to 67.30%)	2.044
PLT/NEU D0 Risk stratification (low vs. intermediate)	0.7266 (0.5876 to 0.8657; *p* = 0.0341)	<62.08	46.15 (36.28% to 56.34%)	100.0 (67.56% to 100.0%)	-
PLT/NEU D0 Risk stratification (low vs. high)	0.7764 (0.6374 to 0.9154; *p* = 0.0107)	<61.68	47.89 (36.68% to 59.31%)	100.0 (67.56% to 100.0%)	-
PLT/NEU D126 Chemoresistance	0.6522 (0.5159 to 0.7884; *p* = 0.0329)	<111.5	57.69 (38.95% to 74.46%)	76.09 (62.06% to 86.09%)	2.413
PLT/NEU D126 Death	0.7766 (0.5247 to 1.000; *p* = 0.0390)	>202.1	80.00 (37.55% to 98.97%)	87.01 (77.72% to 92.79%)	6.160

Abbreviations: AUC: area under the curve; LYM: lymphocytes; NEU: neutrophils; PLT: platelets; “D”: day of treatment.

## Data Availability

The datasets presented in this article are not readily available because the data are part of an ongoing study. Requests to access the datasets should be directed to Dr. Carolina Panis.

## Supplementary Materials

The following supporting information can be downloaded at: https://www.mdpi.com/article/10.3390/medsci13040285/s1. Table S1. Significant hematologic ratios according to the chemoresistance-negative group. Table S2. Significant hematologic ratios according to the negative group for recurrence. Table S3. Significant hematologic ratios according to the metastasis-negative group. Table S4. Significant hematologic ratios according to the negative group for death.
