# Supplementary material for: Leukocyte Ratios Predict Metastasis, Recurrence, and Mortality in Breast Cancer Patients Receiving Cytotoxic Chemotherapy
Source: Med Sci (Basel). 2025 Nov 26;13(4):285. doi: 10.3390/medsci13040285 (PMC12735196; doi:10.3390/medsci13040285)
Supplement: Supplementary file 1 [file medsci-13-00285-s001.zip › medsci-3944003-supplementary.pdf]

Table S1 – Significant hematologic ratios according to the chemoresistance-negative group:

Hematological ratios	Mean 1	Mean 2	Variation	Tukey's test: 95% Confidence Interval; *p*-value	Bonferroni's test: 95% Confidence Interval; *p*-value
LYM/NEU D0 vs. LYM/NEU D42	0.6118	0.981	60%	−0.6925 to −0.04586 $p = 0.0087$	−0.7025 to −0.03583 $p = 0.0107$
MON/LYM D0 vs. MON/LYM D63	0.1406	0.2374	69%	−0.1682 to −0.02543 $p = 0.0003$	−0.1704 to −0.02322 $p = 0.0004$
MON/LYM D0 vs. MON/LYM D84	0.1406	0.2194	56%	−0.1468 to −0.01085 $p = 0.0068$	−0.1489 to −0.008744 $p = 0.0083$
MON/NEU D0 vs. MON/NEU D21	0.07965	0.1321	66%	−0.1035 to −0.001432 $p = 0.0363$	−0.1051 to 0.0001503 $p = 0.052$
MON/NEU D0 vs. MON/NEU D42	0.07965	0.1573	97%	−0.1277 to −0.02756 $p < 0.0001$	−0.1293 to −0.02601 $p < 0.0001$
MON/NEU D0 vs. MON/NEU D63	0.07965	0.1661	109%	−0.1362 to −0.03673 $p < 0.0001$	−0.1377 to −0.03519 $p < 0.0001$
MON/NEU D0 vs. MON/NEU D84	0.07965	0.1444	81%	−0.1121 to −0.01741 $p = 0.0003$	−0.1136 to −0.01594 $p = 0.0003$
NEU/PLT D0 vs. NEU/PLT D147	0.0163	0.00748	−54%	0.001302 to 0.01634 $p = 0.0057$	0.001069 to 0.01657 $p = 0.0068$
PLT/LYM D0 vs. PLT/LYM D42	118.2	186.9	58%	−119.4 to −17.99 $p = 0.0004$	−121.0 to −16.41 $p = 0.0004$
PLT/LYM D0 vs. PLT/LYM D63	118.2	203.6	72%	−135.7 to −34.96 $p < 0.0001$	−137.2 to −33.40 $p < 0.0001$
PLT/LYM D0 vs. PLT/LYM D147	118.2	257.7	118%	−257.7 to −21.17 $p = 0.0052$	−261.4 to −17.50 $p = 0.0062$
PLT/MON D0 vs. PLT/MON D126	1308	2508	92%	−2360 to −41.00 $p = 0.0334$	−2396 to −5.019 $p = 0.0472$
PLT/NEU D0 vs. PLT/NEU D21	67.98	141	107%	−124.7 to −21.24 $p = 0.0001$	−126.3 to −19.64 $p = 0.0001$
PLT/NEU D0 vs. PLT/NEU D42	67.98	168.4	148%	−151.2 to −49.69 $p < 0.0001$	−152.8 to −48.11 $p < 0.0001$
PLT/NEU D0 vs. PLT/NEU D84	67.98	158.7	133%	−138.7 to −42.68 $p < 0.0001$	−140.2 to −41.19 $p < 0.0001$
PLT/NEU D0 vs. PLT/NEU D105	67.98	135.8	100%	−121.7 to −14.05 $p = 0.0016$	−123.3 to −12.38 $p = 0.0018$
PLT/NEU D0 vs. PLT/NEU D126	67.98	151.6	123%	−143.9 to −23.30 $p = 0.0002$	−145.7 to −21.44 $p = 0.0002$

Abbreviations: MON: monocytes; LYM: lymphocytes; NEU: neutrophils; PLT: platelets; "D": day of treatment.

Table S2 – Significant hematologic ratios according to the negative group for recurrence:

				Tukey's test	Bonferroni's test
**Hematological ratios**	**Mean 1**	**Mean 2**	**Variation**	**95% Confidence Interval; *p*-value**	**95% Confidence Interval; *p*-value**
MON/LYM D0 vs. MON/LYM D63	0.1503	0.2368	58%	−0.1501 to −0.02289 $p = 0.0003$	−0.1521 to −0.02092 $p = 0.0004$
MON/LYM D0 vs. MONO/LYM D84	0.1503	0.2301	53%	−0.1395 to −0.02012 $p = 0.0005$	−0.1414 to −0.01827 $p = 0.0005$
MON/NEU D0 vs. MON/NEU D42	0.08402	0.1577	88%	−0.1172 to −0.03030 $p < 0.0001$	−0.1185 to −0.02896 $p < 0.0001$
MON/NEU D0 vs. MON/NEU D63	0.08402	0.165	96%	−0.1251 to −0.03682 $p < 0.0001$	−0.1264 to −0.03546 $p < 0.0001$
MON/NEU D0 vs. MON/NEU D84	0.08402	0.141	68%	−0.09836 to −0.01552 $p = 0.0003$	−0.09964 to −0.01424 $p = 0.0003$
MON/PLT D0 vs. MON/PLT D126	0.001318	0.0006492	−51%	0.0002264 to 0.001112 $p < 0.0001$	0.0002127 to 0.001125 $p < 0.0001$
NEU/PLT D0 vs. NEU/PLT D147	0.01661	0.007355	−56%	0.002862 to 0.01566 $p < 0.0001$	0.002664 to 0.01585 $p < 0.0001$
PLT/LYM D0 vs. PLT/LYM D42	120.2	183.2	52%	−107.8 to −18.19 $p = 0.0002$	−109.2 to −16.81 $p = 0.0002$
PLT/LYM D0 vs. PLT/LYM D63	120.2	209.1	74%	−134.5 to −43.43 $p < 0.0001$	−135.9 to −42.02 $p < 0.0001$
PLT/LYM D0 vs. PLT/LYM D84	120.2	227.8	90%	−150.4 to −64.88 $p < 0.0001$	−151.7 to −63.56 $p < 0.0001$
PLT/LYM D0 vs. PLT/LYM D105	120.2	234.3	95%	−161.7 to −66.61 $p < 0.0001$	−163.2 to −65.14 $p < 0.0001$
PLT/LYM D0 vs. PLT/LYM D126	120.2	236.4	97%	−170.0 to −62.48 $p < 0.0001$	−171.7 to −60.81 $p < 0.0001$
PLT/LYM D0 vs. PLT/LYM D147	120.2	242.5	102%	−227.6 to −17.11 $p = 0.0065$	−230.9 to −13.85 $p = 0.0079$
PLT/MON D0 vs. PLT/MON D126	1289	2528	96%	−2244 to −234.9 $p = 0.0024$	−2276 to −203.8 $p = 0.0027$
PLT/NEU D0 vs. PLT/NEU D21	71.02	142.7	101%	−116.4 to −27.02 $p < 0.0001$	−117,8 to −25,64 $p < 0.0001$
PLT/NEU D0 vs. PLT/NEU D42	71.02	161.6	128%	−133.9 to −47.27 $p < 0.0001$	−135.3 to −45.93 $p < 0.0001$
PLT/NEU D0 vs. PLT/NEU D63	71.02	162	128%	−135.0 to −46.97 $p < 0.0001$	−136.4 to −45.61 $p < 0.0001$
PLT/NEU D0 vs. PLT/NEU D84	71.02	148.8	110%	−119.1 to −36.47 $p < 0.0001$	−120.4 to −35.19 $p < 0.0001$
PLT/NEU D0 vs. PLT/NEU D105	71.02	136,9	93%	−111.8 to −19.86 $p < 0.0001$	−113.2 to −18.44 $p = 0.0001$
PLT/NEU D0 vs. PLT/NEU D126	71.02	142	100%	−123.0 to −18.98 $p = 0.0003$	−124.6 to −17.37 $p = 0.0003$

Abbreviations: MON: monocytes; LYM: lymphocytes; NEU: neutrophils; PLT: platelets; "D": day of treatment.

Table S3 – Significant hematologic ratios according to the metastasis-negative group:

				Tukey's test	Bonferroni's test
**Hematological ratios**	**Mean 1**	**Mean 2**	**Variation**	**95% Confidence Interval; *p*-value**	**95% Confidence Interval; *p*-value**
LYM/PLT D0 vs. LYM/PLT D105	0.01021	0.005088	−50%	0.003389 to 0.006849 $p < 0.0001$	0.003335 to 0.006902 $p < 0.0001$

MON/NEU D0 vs. MON/NEU D84	0.08581	0.1484	73%	−0.1194 to −0.005725 $p = 0.0149$	−0.1212 to −0.003963 $p = 0.0193$
NEU/PLT D0 vs. NEU/PLT D105	0.01652	0.008307	−50%	0.004267 to 0.01217 $p < 0.0001$	0.004144 to 0.01229 $p < 0.0001$

Abbreviations: MON: monocytes; LYM: lymphocytes; NEU: neutrophils; PLT: platelets; "D": day of treatment.

Table S4 – Significant hematologic ratios according to the negative group for death:

				Tukey's test	Bonferroni's test
**Hematological ratios**	**Mean 1**	**Mean 2**	**Variation**	**95% Confidence Interval; *p*-value**	**95% Confidence Interval; *p*-value**
MON/LYM D0 vs. MON/LYM D63	0.1511	0.2414	60%	−0.1490 to −0.03162 $p < 0.0001$	−0.1508 to −0.02980 $p < 0.0001$
MON/LYM D0 vs. MON/LYM D84	0.1511	0.2344	55%	−0.1382 to −0.02831 $p < 0.0001$	−0.1399 to −0.02661 $p < 0.0001$
MON/NEU D0 vs. MON/NEU D21	0.08476	0.1356	60%	−0.09036 to −0.01139 $p = 0.0011$	−0.09158 to −0.01017 $p = 0.0012$
MON/NEU D0 vs. MON/NEU D42	0.08476	0.1624	92%	−0.1159 to −0.03928 $p < 0.0001$	−0.1171 to −0.03810 $p < 0.0001$
MON/NEU D0 vs. MON/NEU D63	0.08476	0.1625	92%	−0.1171 to −0.03848 $p < 0.0001$	−0.1183 to −0.03726 $p < 0.0001$
MON/NEU D0 vs. MON/NEU D84	0.08476	0.14	65%	−0.09207 to −0.01849 $p < 0.0001$	−0.09321 to −0.01735 $p < 0.0001$
PLT/LYM D0 vs. PLT/LYM D42	121.6	186.2	53%	−105.3 to −23.87 $p < 0.0001$	−106.5 to −22.61 $p < 0.0001$
PLT/LYM D0 vs. PLT/LYM D63	121.6	213	75%	−133.1 to −49.60 $p < 0.0001$	−134.4 to −48.31 $p < 0.0001$
PLT/LYM D0 vs. PLT/LYM D84	121.6	230	89%	−147.5 to −69.28 $p < 0.0001$	−148.7 to −68.07 $p < 0.0001$
PLT/LYM D0 vs. PLT/LYM D105	121.6	237.8	96%	−159.9 to −72.43 $p < 0.0001$	−161.2 to −71.07 $p < 0.0001$
PLT/LYM D0 vs. PLT/LYM D126	121.6	237.4	95%	−163.7 to −67.86 $p < 0.0001$	−165.1 to −66.38 $p < 0.0001$
PLT/LYM D0 vs. PLT/LYM D147	121.6	215.4	77%	−177.9 to −9.746 $p = 0.0125$	−180.5 to −7.145 $p = 0.0158$
PLT/MON D0 vs. PLT/MON D126	1284	2409	88%	−1980 to −270.6 $p = 0.0007$	−2007 to −244.2 $p = 0.0008$
PLT/NEU D0 vs. PLT/NEU D21	71.42	134.5	88%	−102.2 to −23.88 $p < 0.0001$	−103.5 to −22.66 $p < 0.0001$
PLT/NEU D0 vs. PLT/NEU D42	71.42	160.1	124%	−126.7 to −50.70 $p < 0.0001$	−127.9 to −49.52 $p < 0.0001$
PLT/NEU D0 vs. PLT/NEU D63	71.42	159.2	123%	−126.7 to −48.74 $p < 0.0001$	−127.9 to −47.53 $p < 0.0001$
PLT/NEU D0 vs. PLT/NEU D84	71.42	147.6	107%	−112.7 to −39.67 $p < 0.0001$	−113.8 to −38.54 $p < 0.0001$
PLT/NEU D0 vs. PLT/NEU D105	71.42	133	86%	−102.5 to −20.78 $p < 0.0001$	−103.7 to −19.52 $p < 0.0001$

PLT/NEU D0 vs. PLT/NEU D126	71.42	138.6	94%	−112.0 to −22.46 $p < 0.0001$	−113.3 to −21.08 $p < 0.0001$

Abbreviations: MON: monocytes; LYM: lymphocytes; NEU: neutrophils; PLT: platelets; "D": day of treatment.
